# Developing knowledge, attitude and practice questionnaire in desmoid tumours (KAPID study): applying e-Delphi to rare diseases

**DOI:** 10.3332/ecancer.2024.1715

**Published:** 2024-06-20

**Authors:** Ghazal Tansir, Sameer Rastogi, Simran Kaur, Mrinal Gounder, Soumya Muta

**Affiliations:** 1 Department of Medical Oncology, All India Institute of Medical Sciences, New Delhi 110029, India; 2 Department of Physiology, All India Institute of Medical Sciences, New Delhi 110029, India; 3 Department of Medical Oncology, Memorial Sloan Kettering Cancer Center, Weill Cornell Medical College, New York, NY 10065, United States of America; 4 Department of Internal Medicine, All India Institute of Medical Sciences, New Delhi 110029, India

**Keywords:** e-Delphi, rare cancers, desmoid tumour, questionnaire

## Abstract

**Background:**

Rare diseases are associated with unique challenges encountered in diagnosis, treatment and conduct of clinical research. Desmoid tumour (DT) is one such ultra-rare malignancy about which awareness among medical professionals remains limited. We developed a questionnaire to assess knowledge, attitude and practice (KAP) among medical professionals on DT.

**Methods:**

E-Delphi method was used for the assessment of KAP for DT amongst clinical experts (experience of >/= 3 years in DT). 22 open-ended statements were developed by the core research group using current consensus guidelines. In round 1, experts provided subjective feedback which was incorporated into a 35-item questionnaire. Round 2 entailed experts giving feedback as a 5-point Likert scale classified into agreement (median score >/=4), neutral (median score 3) and disagreement (median score <3). Feedback from Round 2 was incorporated and questions with neutral consensus were modified. Questions in Round 3 achieved consensus if >/= 75% participants agreed.

**Results:**

11 (64.7%) of 17 contacted experts responded in Round 1 including 6 (54.4%) who gave additional inputs and 5 (45.6%) who agreed to all statements. In round 2, 8 out of 11 experts responded to the 35-item questionnaire on knowledge (*n* = 16), attitude (*n* = 8) and practice (*n* = 11). 32 questions obtained agreement and 3 (8.5%) had neutral consensus. These were modified for round 3, in which consensus on 2 (66.6%) was attained. The final questionnaire comprises 34 items with 15, 8 and 11 questions on in the sections of knowledge, attitude and practice (KAP), respectively.

## Background

Rare diseases are defined as diseases with a low prevalence in the general population, defined as less than 5 per 10,000 inhabitants [1]. Rare malignancies are defined as malignancies with an incidence rate of less than 6 per 100,000 population [2]. Given that patients with rare diseases are scattered across healthcare centers, unique challenges are encountered in their diagnosis and management. These include a lack of adequate sample size to study the natural history of the disease, varied clinical presentations, delayed or incorrect diagnosis, lack of access to specific therapies and expert centres, and unavailability of relevant clinical trials [3].

Desmoid tumour (DT) is an ultra-rare, locally aggressive disease with an annual incidence of 5–6 cases per million population [4]. Not all cases of DT require systemic therapies and active surveillance can lead to spontaneous regression in approximately 30% patients [5]. Systemic treatment modalities for DT have evolved over the past years with the disuse of tamoxifen and non-steroidal anti-inflammatory drugs and greater use of tyrosine kinase inhibitors (TKIs) and the recently approved nirogacestat, a gamma secretase inhibitor [6, 7].

Awareness among medical professionals regarding the diagnosis and treatment options for DT remains limited and there is a global consensus to refer patients to centres with sarcoma expertise. Despite a decade of evidence against the routine use of surgery, this practice still continues and results in significant morbidity [8]. Previous studies have demonstrated the lacuna in the inclusion of rare diseases in current medical curricula, with the prevailing lack of awareness about such conditions among medical students [9]. This could translate into mitigating the awareness deficits among diagnosticians and treating clinicians.

We have developed a questionnaire targeting physicians and trainee doctors involved in the management of DT to assess their KAP about the disease. This will include medical professionals currently pursuing postgraduate residency in medical and surgical branches. We utilised the e-Delphi method which enables building consensus on subjects that have minimal existing information [10]. A questionnaire that can comprehensively incorporate information on a rare condition such as DT would require inputs from a very specific cohort of experts. Thus, the e-Delphi method was used to collate the opinions of this cohort through a structured communication process without the experts requiring geographical proximity [11].

## Methods

The KAP assessment questionnaire was developed at the All India Institute of Medical Sciences, New Delhi using the e-Delphi method. The e-Delphi technique is the electronic version of the traditional Delphi method, which was originally developed by the Rand corporation in the 1950s to collect data from participants on a paper-based questionnaire [12, 13]. The aim of the Delphi method is to consolidate group diversity into an expert opinion, this method allows a group consensus on a topic of interest [14]. Conducting and REporting DElphi Studies (CREDES) guidelines were followed for CREDES [15]. The purpose of the study was to achieve a consensus on questions to be included in a KAP assessment tool among medical professionals for DT.

**Figure 1.**
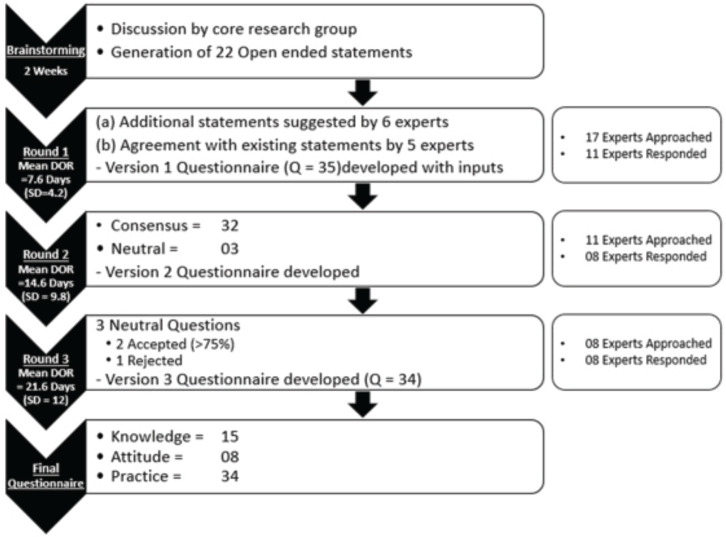
Flow and timeline of development of KAP assessment questionnaire by the e-Delphi method. KAP: knowledge, awareness, practice; DOR: duration of response, SD: standard deviation.

The iterations were carried out in the traditional manner over a sequence of three rounds which were moderated by one common member of the core research group. The steps of the questionnaire development are described as follows (Figure 1).

### Phase 1 (Preparation)

#### Formation of core group

The core group for conducting the study comprised four members including a faculty and post-doctoral fellow from the Department of Medical Oncology, a faculty from the Department of Physiology and a postgraduate resident from the Department of Medicine. The core group was formed keeping in view the intellectual, methodological and statistical expertise required for the conduct of the Delphi-based study.

#### Preparatory research

A thorough research was carried out on existing literature on DT including clinical presentation, diagnosis and treatment options. Literature was also reviewed to formulate the structure in which the study was to be conducted. It was thus decided to carry out the study by the sequential, e-Delphi technique. The definitions of KAP were specified as below:

Knowledge: Assessment of the extent to which the knowledge of participants corresponds to existing biomedical concepts [16].

Attitude: A learned predisposition to think, feel and act in a particular way towards the topic [17].

Practice: The use of preventative measures or different health options in the management of diseases [18].

### Selection of experts

The number of experts deemed adequate for the conduct of the study was decided as 10 as per previous recommendations [19]: An initial number of at least 17 experts was planned to be approached keeping in view an expected dropout rate of 30%. To be classified as an expert, the individual should have been working actively as a medical professional in the field of DT for a minimum of 3 years. Steps for selection of these experts were identifying the names of experts, ranking them based on qualifications, inviting the individuals, giving them the option to nominate others for inclusion into the study and confirming their commitment to participation in this multi-round study.

### Formulation sessions

Discussion sessions among the core group members were carried out to create open-ended statements on the subject of DT covering causative factors, pathologic features and management. 22 open-ended statements were formulated to be shared as part of the first round of the Delphi method and divided into knowledge (*n* = 8), attitude (*n* = 11) and practice (*n* = 3) sections.

### Phase 2 (Conduct of the study by Delphi method)

#### Round 1 (semi-structured)

The experts were contacted via e-mail and informed about the rationale and specifics of the study. The open-ended statements proposed during the planning round by our core research group were provided. The experts were requested to revert with subjective feedback on each statement regarding relevance for inclusion into the questionnaire and to provide additional statements that could be included. The respondents were given 2 weeks for responding in Round 1 and a reminder email was sent in case of no response at 2 weeks.

#### Round 2 (structured)

The open-ended feedback was summarised in a single document and structured into the sections of knowledge (*n* = 16), attitude (*n* = 8) and practice (*n* = 11). The statements that were agreed upon or suggested by the experts were converted into multiple-choice questions. A 35-item multi-choice questionnaire (Version 1) was designed based on the suggestions obtained during the first round. The questionnaire was mailed to the experts who responded in the first round with a 5-choice Likert scale. The experts were asked to rate their agreement with the inclusion of the question into the questionnaire as 5 (Strongly agree), 4 (Agree), 3 (Neutral), 2 (Disagree) and 1 (Strongly disagree). Each question also had a column for additional feedback comments to be given by the experts. The decision to incorporate this additional feedback was the prerogative of the core research group.

#### Round 3 (structured)

Questions that did not meet the consensus of the experts were modified as per the comments provided in the feedback section. Questions that had reached consensus also underwent slight modifications as per the additional feedback provided. This Version 2 of the questionnaire was sent to the experts for review. The inputs required were to be in the form of ‘Accepted’ or ‘Not accepted’ to the modified questions that had not reached consensus in Version 1. A response period of 2 weeks each was provided in Rounds 2 and 3 and a reminder email was sent after 1 week.

#### Statistical analysis

Definitive item selection criteria were specified from Round 2 by interpretation of the contingency table. Consensus was defined as a median rating score of >/= 4; non-consensus was classified as neutral (median score 3–4) and disagreement (median score <3) (Table 1). Questions that reached ‘neutral’ consensus were modified according to suggestions provided in the qualitative feedback given by the experts. The modified questionnaire was sent to the experts for assessment in Round 3. In Round 3, the experts were specifically required to respond ‘accepted’ or ‘not accepted’ for the modified questions.

## Results

Round 1: 17 experts were contacted with 22 open-ended statements proposed during the brainstorming session of the core research group (Appendix). Characteristics of experts who were involved in at least one round of development of the questionnaire are detailed in Table 2. Out of 17, 11 (64.7%) experts responded with a mean response time of 7.6 days (standard deviation = 4.2). 6 (54.5%) experts gave inputs in the form of statements to be included in the questionnaire and 5 (45.4%) reverted that they agreed with the contents and did not provide further suggestions. 6 (54.5%) did not provide any response and were excluded from the further process (Figure 1).

Round 2: The 35-item questionnaire (version 1, Supplementary 2) was sent to 11 experts, of whom 8 (72.7%) responded with a mean response time of 14.6 days (standard deviation = 9.8). 14 (87.5%) items in knowledge, 8 (100%) in attitude and 10 (90.9%) in practice sections, respectively, obtained the consensus agreement (Table 3). 3 (8.5%) questions had neutral consensus while none had a disagreement. The modification was made to the questions with neutral consensus based on the median scores and qualitative feedback, to build version 2.

Round 3: The modified questionnaire (version 2, Supplementary 3) was sent to eight experts, all of whom (100%) responded with a mean response time of 21.6 days (standard deviation = 12.0). Three questions required scoring to be repeated by experts. 62.5%, 87.5% and 75% acceptance was reached for question numbers 5, 6 and 29, respectively. The final questionnaire (version 3, Table 4) consists of 34 items consisting of 15, 8 and 11 questions in KAP sections, respectively.

## Discussion

In this study, we describe the process of development of a questionnaire to assess the KAP of medical professionals regarding DT. The e-Delphi method was employed in our study because of the advantage of faster response times and inexpensive functioning. The experts with clinical and research interests in rare malignancy such as DT were situated in different geographic areas. Conduct of multiple rounds of feedback to achieve a group agreement could be performed over a limited time period using electronic media [20]. e-Delphi method has been used in the area of healthcare research in previous studies [21] and for the first time in the domain of DT.

KAP assessment questionnaires have been developed and used in highly prevalent malignancies such as cervical, prostate and gastrointestinal cancers [22–24]. A Chinese cross-sectional study among healthcare workers found moderate knowledge and positive attitudes regarding early gastrointestinal cancers [24]. The presence of good knowledge and a positive attitude were found to correlate with excellent practice in this survey. Such tools are scarcely utilised in rare diseases and no such assessment questionnaires have been utilised specifically for DT so far. A survey on awareness about rare diseases among medical doctors pursuing residency found that 94.6% perceived their knowledge as insufficient or very poor despite 83% acknowledging that rare diseases were a serious public health issue [25]. Moreover, it has been demonstrated that the inclusion of newer education tools and topics into medical curricula may translate into improved patient care [26]. We therefore aimed to develop a KAP assessment tool targeting DT, which is an ultra-rare disease with challenging diagnosis and management.

The diagnosis and treatment of DT requires expertise in its pathologic findings as well as optimum management. The use of extensive surgeries and cytotoxic chemotherapy has been observed in a cross-sectional study conducted at our centre [27]. The study found that 7.8% of patients fulfilled the clinical criteria of major depressive disorder along with lower global QoL scores in the DT cohort as compared to historical controls. The physical and psychological morbidity caused by this disease could potentially be mitigated by better physician understanding of various aspects of DT. Thus, this questionnaire was developed by our research team as a starting point to assess the KAP among medical professionals.

During the steps of the development of this questionnaire, the only question that did not gain final acceptance despite modifications was based on superficial versus deep DT. Thus, all but one question gained acceptance by the expert panel. Recognising the paucity of expert voices in this field and the known rates of attrition per round noted in Delphi-based studies [28], we made modifications to the development design. We adopted the consensus cut-off of 75%, which has been validated in previous studies for the final round instead of the scoring criteria [29]. We could thereby obtain 100% response rates in the final round which strengthened the results of the study.

The strengths of our study include the first development of a KAP assessment tool for an ultra-rare malignancy, inclusion of experts on a global platform and high response rates for each round of development. While 10–18 experts have been recommended in previous studies [14], the number of experts who actively participated in the development of our questionnaire is optimum in this rare condition. By using the e-Delphi method, we were able to circumvent the drawbacks of face-to-face meetings such as domination by strong personalities and normative conformity pressures [30]. The e-Delphi technique can also be replicated in developing assessment tools pertaining to other rare diseases.

The development of the questionnaire was encountered with multiple challenges. The field of DT has few experts because of the rarity of the disease. Thus, it was difficult to find experts with the requisite experience in this niche field. Despite the convenience involved in the e-Delphi method, there were restrictions in extracting responses in a time-bound fashion when the experts are not available in person.

### Future directions

This questionnaire can be used to assess medical professionals to gauge their prevailing deficits in KAP pertaining to DT. Validation of the questionnaire will be performed among a cohort of 30 resident doctors in training in departments of medical oncology, surgical oncology, radiation oncology and general surgery. Following the validation, this tool can be implemented in other referral and peripheral centres to yield differences in the level of awareness that medical professionals possess. The knowledge obtained can be used to develop strategies to better apprise medical professionals about necessary aspects of DT. The technique of e-Delphi method can also be utilised for creating tools for other rare malignancies with a limited pool of experts. This KAP model may be utilised for building a questionnaire based on different rare diseases. The e-Delphi technique can serve as a vital tool for including experts of different rare diseases who are situated across different health centres.

## Conclusion

We have designed a questionnaire to assess the KAP on DT in medical professionals using the e-Delphi method. Through this study, we emphasise the importance of assessment tools for rare malignancies and how the e-Delphi method can be an efficient method for their development. The 34-item questionnaire can be utilised at multicentre levels for studies after initial validation.

## List of Abbreviations

DT, Desmoid tumour; KAP, Knowledge, attitude, practice; TKI, Tyrosine kinase inhibitor; Conducting and REporting DElphi Studies.

## Conflicts of interest

No conflict(s) of interest exist for the authors.

## Funding

Mrinal Gounder, MD receives personal or research funding from Springworks Therapeutics, Ayala and Iterion.

No funding has been received from any organisation for this submitted work.

No recent, present or anticipated employer will gain or lose financially through publication of this manuscript.

No stocks or shares in companies will gain or lose financially through publication of this manuscript.

There are no additional non-financial interests to declare.

## Author contribution

GT, SR, SK and SM formed the core research group and participated in each step of development of the questionnaire. All authors performed preparatory literature search. GT contacted the experts and moderated the rounds of questionnaire development. SR and SK supervised the conduct of the study. GT developed the manuscript. SR, SK and MG supervised and corrected the study manuscript. All authors reviewed and approved of the final version of the manuscript.

## Ethics approval

The study was approved by the Institute Review Board (IECPG-474/24.08.2023) and was performed in accordance with the principles of the 1964 Declaration of Helsinki.

**Interpretation:** The resulting 34-item questionnaire was developed by the e-Delphi method for a rare tumour like DT. This questionnaire may be used as a tool in DT and other rare diseases.

## Clinical trial registration

Not applicable.

## Data availability statement

All the data supporting the findings of this study are available within the paper and its Supplementary Information.

## Figures and Tables

**Table 1.** Consensus criteria for inclusion of questions based on responses of experts.

Consensus	Score per question
Agreement	Median >/=4
Neutral	Median 3-4
Disagreement	Median <3

**Table 2.** Characteristics of members of the expert panel who responded to at least one round of the questionnaire development.

Characteristics	Details
Gender	Male (*n* = 10, 90.9%) Female (*n* = 1, 9.1%)
Country of work	North America (*n* = 5, 45.4%) India (*n* = 4, 36,3%) United Kingdom (*n* = 1, 9.1%) Germany (*n* = 1, 9.1%)
Primary specialty	Medical oncology (*n* = 6, 54.5%) Surgical oncology (*n* = 3, 27.2%) Pediatric oncology (*n* = 1, 9.1%) Orthopaedics (*n* = 1, 9.1%)
Mean years of experience in DT	11.2 years (Standard deviation = 3.03)

**Table 3.** Contingency table of responses obtained to version 2 of the questionnaire.

Question number	Mean	Standard error mean	Median	Interquartile range	Variance
1	3.71	0.47	4	2	1.83
2	4.75	0.16	5	1	0.21
3	4.12	0.47	5	1	1.83
4	4.62	0.26	5	0	0.554
5	3.12	0.35	3	1	0.98
6	3.37	0.49	3.5	2	1.98
7	4.87	0.12	5	0	0.12
8	3.85	0.25	4	1	0.47
9	4.71	0.18	5	1	0.23
10	4.37	0.37	5	1	1.12
11	4.37	0.18	4	1	0.26
12	4.62	0.18	5	1	0.26
13	3.3	0.37	4	1	1.12
14	3.57	0.57	4	3	1.28
15	4.25	0.59	4	4	1.78
16	4.37	0.37	5	1	1.12
17	4	0.37	4	1	1.00
18	4.14	0.40	4	1	1.14
19	4.85	0.14	5	0	0.14
20	4.57	0.20	5	1	0.28
21	4.42	0.20	4	1	0.28
22	4.14	0.55	5	1	2.14
23	4.3	0.62	4	3	2.12
24	4	0.46	4	1	1.71
25	4	0.37	4	2	1.0
26	4.28	0.42	5	1	1.23
27	4.57	0.48	4	3	1.61
28	3.71	0.28	4	1	0.57
29	3.28	0.52	3	3	1.90
30	4.87	0.12	5	0	0.12
31	3.87	0.47	4	2	1.83
32	4.87	0.12	5	0	0.12
33	4.85	0.14	5	0	0.14
34	4.75	0.16	5	1	0.21
35	4.14	0.26	4	1	0.47

**Table 4.** Final version of questionnaire developed for assessment of knowledge, assessment and practice among medical professionals on DTs.

No.	Questions
1.	Where have you received your knowledge on DTs? (multiple answers) a. Classes during residency/fellowship b. Scientific literature c. Conferences or symposia d. Others (specify)
2.	The prevalence of DTs can best be described as: a. Common b. Rare c. Ultra-rare d. Not sure
3.	The age group most frequently affected by DTs is: a. Pediatric (0–15 years) b. Adolescents and young adults (15–39 years) c. Adults (39–65 years) d. Geriatric (>65 years) e. Not sure
4.	The gender distribution among patients with non-FAP related DT is best described as: a. Male preponderance b. Female preponderance c. Equal distribution d. Not sure
5.	Factors associated with development or exacerbation of DTs include (multiple answers): Pregnancy Surgery Trauma Radiation Not sure
6.	Most frequent gene mutation in DTs is a. BRAF b. P53 c. CTNNB1 d. AKT1 e. Not sure
7.	Pathways leading to development of DT include (multiple answers): a. Somatic mutations b. Germline FAP mutations c. Epigenetic mutations d. Not sure
8.	The most commonly reported sites of DTs include (multiple answers): a. Trunk b. Viscera c. Extremities d. Abdominal wall e. Not sure
9.	Syndrome associated with risk of development of DTs include: a. Gardner/FAP syndrome b. Turcot syndrome c. Li Fraumeni syndrome d. Muir Torre syndrome e. Not sure
10.	The biological behaviour of DTs can best be described as: a. Benign b. Locally aggressive c. Malignant d. All of the above e. Not sure
11.	The pattern of spread of DT can best be described as: a. Localised b. Locally infiltrative c. High frequency of distant metastases d. All patterns equal e. Not sure
12.	The most predominant clinical features which make you suspect DT (multiple answers): a. Slow growth of tumor b. Solid palpable mass on clinical exam c. Fever d. Pain e. Not sure
13.	Estimated 5-year recurrence rate after first surgery for DT is: a. <25% b. 25%–50% c. 50%–75% d. More than 75% e. Not sure
14.	The approximate proportion of asymptomatic patients with DT that may progress on active surveillance is: a. 20% b. 40% c. 60% d. 80% e. Not sure
15.	Non-surgical interventions for management of DTs that you are aware of (multiple answers): a. Radiofrequency ablation b. Transcatheter arterial chemoembolisation c. Cryotherapy d. Radiotherapy e. Not sure
16.	Rate your preparedness for managing a patient with DT on a scale of 0–5 with 5 being the best score 0 1 2 3 4 5
17.	Rate your agreement on DT being part of undergraduate and/or postgraduate teaching on a scale of 0–5 with 5 being the best score 0 1 2 3 4 5
18.	Rate your agreement on assessment of quality of life being part of DT management on a scale of 0–5 with 5 being the best score 0 1 2 3 4 5
19.	Radiological investigation of choice for local imaging of DT is: a. Computed tomography b. Magnetic resonance imaging c. Ultrasonography d. Any of the above e. Not sure
20.	Specialists who should be trained in management of DTs includes (multiple answers): a. General physicians/surgeons b. Intervention radiologists c. Medical oncologists d. Surgical oncologists e. All of the above
21.	Surgical management of DTs is done in the following (multiple answers): a. At first diagnosis b. Progressive disease post-medical or radiation therapy c. Vital organ compromise due to tumor d. Role of surgery is extremely limited e. Not sure
22.	Sub-sites of DT which may require multiple surgical procedures include (multiple answers): a. Extremity b. Abdominal wall c. Intra-abdominal d. Depends on the clinical presentation e. Not sure
23.	Subset of patients in which screening colonoscopy would be warranted includes (multiple answers): a. All patients with DTs b. Family history of colorectal cancers c. Family or personal history of FAP/Gardner syndrome d. Depending on site of the tumor e. Not sure
24.	The number of patients with DT you have come across in the past year: a. <10 b. 10–50 c. 50–100 d. >100 e. Not sure
25.	You request the pathologist for immunohistochemistry markers for diagnosis of DT in the following: a. All cases b. Only cases not diagnosed morphologically c. Never d. Not sure
26.	You request the pathologist for hormonal receptor status before starting treatment for which patients with DT: a. All cases b. Patients planned for hormonal therapy c. Never d. Not sure
27.	In which subset of patients with DTs should germline mutation testing be performed (multiple answers): a. All patients b. Patients with significant family history c. Based on somatic mutation profile d. For patients without any obvious risk factors e. Not sure
28.	Next generation sequencing is included in management of DTs in your practice (multiple answers): a. For all patients b. Based on family history or histopathology c. Unavailability of RT PCR for CTNNB1 or APC mutation d. Never e. Not sure
29.	You discuss patients with DTs in multidisciplinary meetings in your centre (multiple answers): a. All patients b. Only if planning surgery or radiotherapy c. Post multiple-lines of therapy d. Never e. Not sure
30.	If a patient who had undergone initial surgical resection now presents to you with symptomatic disease recurrence, which would be your preferred therapy (multiple answers): a. Repeat surgery b. Targeted therapy c. Chemotherapy d. Radiotherapy e. Active surveillance f. Not sure
31.	Your preferred treatment option(s) for asymptomatic or minimally sympatomatic patients with extremity DTs (multiple answers): a. Surgery b. Targeted therapy c. Chemotherapy d. Active surveillance e. Radiotherapy f. Not sure
32.	Your preferred treatment option(s) for minimally symptomatic patients with extremity DTs on hormonal therapy with documented radiological progression in serial scans (multiple answers): a. Surgery b. Targeted therapy c. Chemotherapy d. Active surveillance e. Not sure
33.	Your preferred treatment option(s) for symptomatic patients with extremity DT include (multiple answers): a. Surgery b. Targeted therapy c. Chemotherapy d. Active surveillance e. Radiotherapy f. Not sure
34.	The most frequently used systemic therapies at your centre for DTs/fibromatosis include (multiple answers): a. Hormonal therapy b. Anti-inflammatory therapy c. TKIs d. Chemotherapy e. Novel therapies from clinical trials f. Not sure

## Appendix: Brainstorming ideas on questionnaire development to assess knowledge attitude practice among healthcare professionals for desmoid tumor

### 1. Knowledge

The respondent can be asked about the following aspects:

1. Whether they are aware about the term desmoid/fibromatosis
2. Where they got their knowledge about desmoid from and whether they were taught about desmoid tumors during their studies.
3. How prevalent do they think desmoid is and the age group of patients it usually occurs in
4. Gender distribution of desmoid tumors
5. About the sporadic or inherited nature of the disease
6. The nature of desmoid - whether benign or malignant
7. The usual quality of life of patients living with desmoid tumors
8. Knowledge about 5-year recurrence rates for desmoid after first surgery

### 2. Attitude

The respondent can be asked about the following aspects:

1. Perception on their knowledge about desmoid tumor by rating their knowledge
2. Perception on whether they feel fully prepared to care for a patient with desmoid tumor
3. Willingness to increase their knowledge about desmoid tumors
4. Which specialities should be trained for management of desmoid tumors
5. Whether there should be a mandatory course on desmoid/fibromatosis in medical curricula?
6. Types of radiological investigations best suited for diagnosis of desmoid tumors
7. Treatment approach recommended for asymptomatic patients with desmoid tumors - targeted therapy, surgery, chemotherapy, surveillance
8. Treatment approach recommended for mildly symptomatic patients with desmoid tumors
9. When surgery should be considered as part of treatment of desmoid
10. Whether disabling extensive procedures have a role in surgical management of desmoid tumors
11. Importance of patient related outcomes in management of desmoid tumors

### 3. Practice

The respondent can be asked about the following aspects:

1. The radiological investigation that they prefer for diagnosis of desmoid tumors
2. Whether or not they use immunohistochemistry markers or hormonal receptors for diagnostic and therapeutic purposes
3. Whether they prefer to operate the desmoid tumor after the first recurrence
